# Characterization and clinical implications of ankle impedance during walking in chronic stroke

**DOI:** 10.1038/s41598-021-95737-6

**Published:** 2021-08-18

**Authors:** Amanda L. Shorter, James K. Richardson, Suzanne B. Finucane, Varun Joshi, Keith Gordon, Elliott J. Rouse

**Affiliations:** 1 Department of Biomedical Engineering, McCormick School of Engineering, Northwestern University, Evanston, IL USA; 2 The Center for Bionic Medicine, Shirley Ryan AbilityLab, Chicago, IL USA; 3 Department of Physical Medicine and Rehabilitation, University of Michigan, Ann Arbor, MI USA; 4 Department of Mechanical Engineering, University of Michigan, Ann Arbor, MI USA; 5 Department of Physical Therapy and Human Movement Sciences, Feinberg School of Medicine, Northwestern University, Chicago, IL USA; 6 Edward Hines Jr. Veterans Affairs Hospital, Hines, USA; 7 Core Faculty, Robotics Institute, The University of Michigan, Ann Arbor, MI USA

**Keywords:** Biomedical engineering, Mechanical engineering, Neurology

## Abstract

Individuals post-stroke experience persisting gait deficits due to altered joint mechanics, known clinically as spasticity, hypertonia, and paresis. In engineering, these concepts are described as stiffness and damping, or collectively as joint mechanical impedance, when considered with limb inertia. Typical clinical assessments of these properties are obtained while the patient is at rest using qualitative measures, and the link between the assessments and functional outcomes and mobility is unclear. In this study we quantify ankle mechanical impedance dynamically during walking in individuals post-stroke and in age-speed matched control subjects, and examine the relationships between mechanical impedance and clinical measures of mobility and impairment. Perturbations were applied to the ankle joint during the stance phase of walking, and least-squares system identification techniques were used to estimate mechanical impedance. Stiffness of the paretic ankle was decreased during mid-stance when compared to the non-paretic side; a change independent of muscle activity. Inter-limb differences in ankle joint damping, but not joint stiffness or passive clinical assessments, strongly predicted walking speed and distance. This work provides the first insights into how stroke alters joint mechanical impedance during walking, as well as how these changes relate to existing outcome measures. Our results inform clinical care, suggesting a focus on correcting stance phase mechanics could potentially improve mobility of chronic stroke survivors.

## Introduction

Stroke is the leading cause of adult-onset disability, affecting millions of Americans^1^; however, treatment of the locomotor dysfunction that often results has been limited. Up to 80% of stroke survivors experience persistent gait deficits even after standard rehabilitation therapies^2,3^ that increase their risk for recurrent stroke and vascular death^4^. Changes in joint kinetics, kinematics and mechanics manifest as reduced gait speed, instability, asymmetry, and exhaustion^2,3^. While kinetic and kinematic impairments during walking have been studied extensively, altered joint mechanics (stiffness and damping) are poorly understood. Following stroke, the mechanical properties of muscle are fundamentally altered, causing an increase in passive and active muscle tone, as well as altered reflex facilitation and inhibition^5^. Clinically, changes to joint stiffness and damping are referred to as spasticity, hypertonia, and paresis^5,6^. Spasticity is thought to be a velocity dependent resistance to movement^7^. Hypertonia and co-activation can cause an increase in joint stiffness, while limb paresis can cause a decrease in joint stiffness^8^. To assess altered mechanics and their effects on mobility, clinicians often use coarse, qualitative assessments during non-weight bearing or resting conditions (*e.g.* Modified Ashworth Scale). These assessments are used to help guide treatment, and although current physical therapy and pharmacological treatments have been successful in passive conditions^9,10^, this success has not translated to functional improvements in locomotion^5^. Lack of improvement may stem from fundamental differences in how altered joint mechanics present between passive movement and dynamic movement. Furthermore, although stiffness and damping are well defined properties in engineering, their clinical analogs are not as distinct. Spasticity, hypertonia, and paresis are all changes to joint mechanics, but their clinical characterizations are broad and do not map to any specific impedance property (stiffness or damping). Directly characterizing altered joint stiffness and damping during walking may supplement the qualitative assessment metrics used in the clinic, while reconciling the mismatch between passive assessment and desired improvements in dynamic activities.

Joint stiffness and damping (along with inertia) are collectively known as joint *mechanical impedance*, and are fundamental to our ability to regulate interaction with the environment. There is evidence that the mechanical impedance of limbs helps provide stability during unstable tasks and compensates for unexpected environmental dynamics^11–13^. Biped walking is inherently mechanically unstable, and is one common task where joint stiffness and damping may play an important role in stability and forward propulsion. In young, healthy adults, ankle joint stiffness and damping vary continuously throughout the stance phase of walking^14–16^. The stiffness component of impedance increases during loading response (early stance phase), mid-stance, and early terminal stance phase in preparation for push off^14^, then decreases to values reported in swing throughout late terminal stance as the heel rises^15,16^. Ankle joint damping values in young healthy adults remain constant throughout loading response, mid-stance, and early terminal stance phase of walking, and increased during late terminal stance as the heel rises^14,16^. Characterizing ankle joint impedance in young healthy adults during gait has improved our understanding of gait biomechanics and led to the design of novel biomimetic prosthetic devices^17,18^. However, knowledge of how joint impedance is altered following neurological injury (e.g. hemiparetic stroke) is limited, and therefore has not yet been incorporated into treatment strategies and standards of care.

Previous research into altered joint impedance following hemiparetic stroke has focused on static conditions, rather than dynamic tasks, such as walking. These studies show that stiffness of the affected ankle in static conditions is significantly increased in stroke survivors^19,20^, but the component of stiffness associated with reflexes was not found to be different between groups^21^. These increases in ankle stiffness are not consistent throughout the population with some participants showing no difference in passive ankle stiffness from controls without a history of neurological injury^22,23^. Characterization of the damping component of impedance has been limited, but Mirbagheri et al*.* found no significant difference between paretic and non-paretic limbs under static conditions^20^. Investigations of the relationship between joint impedance and kinetic and kinematic properties of the ankle have also been conducted. Under static conditions, joint impedance been shown to be related to muscle activity^24–26^, ankle position^27–29^, and ankle torque^24,30^. In seated static conditions, stiffness was found to increase proportionally with mean ankle torque^24,30^, increase with activation and co-activation of the tibialis anterior and soleus muscles^25^, and increase with degree of planterflexion or dorsiflexion^27,29,31^. These relationships appear to hold in individuals with chronic stroke under similar static conditions^19,20^, however, as seen in studies on unimpaired populations, it is increasingly unlikely that the relationships between stiffness, joint torque, and muscle activation are maintained under dynamic conditions^32^. Therefore, although these works have provided valuable insight into how joint impedance is altered post-stroke and the relationship to kinetic and kinematic factors, their insights should not be extended to dynamic tasks such as locomotion.

The purpose of this study was to (1) estimate impedance of the ankle joint during walking in individuals with chronic stroke, (2) characterize the relationship between ankle impedance and muscle activity, and (3) investigate the relationship between impedance impairment and clinical measures of mobility, spasticity, and sensorimotor function. Our primary hypothesis was that joint stiffness of the paretic limb would be increased during loading response (early stance phase), where muscle activity at the ankle is limited and increased passive joint stiffness dominates^33,34^, and decreased during mid-stance and early terminal stance due to reduced muscle activation^35^. It was also hypothesized that standard clinical measures of mobility would correlate with impedance impairment during walking, but clinical measures of impairment obtained passively would not. This work provides a foundation for a new assessment paradigm where the factors guiding treatment such as orthotic bracing, pharmaceutical management or physical therapy, can be directly measured quantitatively, rather than inferred from coarse, qualitative studies at rest. Furthermore, these results could inform new clinical targets for therapeutic interventions and the development of novel assistive technologies that leverage knowledge of altered joint mechanical impedance during gait. Preliminary results for this work with a subset of participants was presented at the 2019 International Conference on Rehabilitation Robotics^36^.

## Results

### Stiffness and damping estimates

A second order parametric model described how the perturbation induced displacements corresponded to the resultant torque response needed to estimate mechanical impedance. The second order model characterized ankle mechanical impedance during walking in participants with chronic stroke with Variance Accounted For (VAF) of 75% ± 14% for the paretic limb and 75% ± 13% for the non-paretic limb. The model fit is notably lower than age-range matched older adults (VAF = 97 ± 3%) and previous studies^14,16^. The contributions of stiffness, damping, and inertia to the resultant torque were 30.3% ± 17.7%, 9.72% ± 8.73%, and 26.9% ± 15.8% respectively for the paretic limb. For the non-paretic limb stiffness contributed 34.7% ± 18.1% of resultant torque, damping contributed 7.81% ± 5.58%, and inertia contributed 22.7% ± 15.9%.

**Figure 1:**
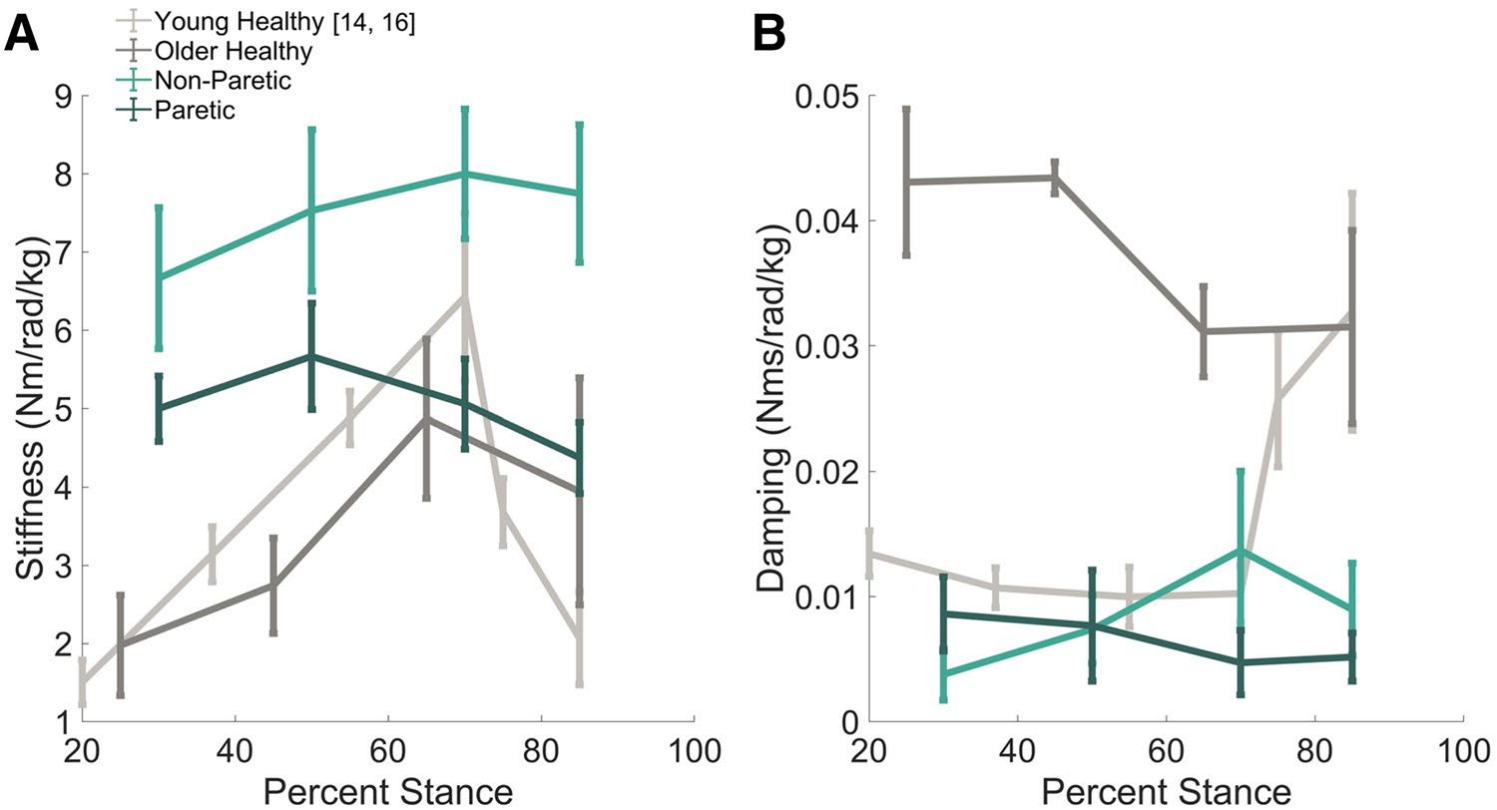
Average inter-subject stiffness (**A**) and damping (**B**) as a function of stance phase. Ankle impedance estimates during walking of individuals with chronic stroke are indicated in dark green (paretic limb) and light green (non-paretic limb). Dark grey traces indicates impedance estimates of three gait-speed matched older adults without stroke, within a similar age range to participants with chronic stroke. Light grey traces present impedance as a function of stance phase for young healthy adults walking at a faster speed from previous literature. Stiffness for stroke participants was constant across the stance phase of walking and did not demonstrate the stereotypical increase in mid-stance that prepares for forward propulsion for either limb. Stiffness of the non-paretic limb was significantly larger than the paretic limb, and both were increased compared to age and gait-speed matched controls. Older adults walking at a slower pace exhibited a similar pattern of stiffness variation to young healthy adults with a lower peak stiffness in mid-stance. Damping did not vary significantly across stance phase for either limb of stroke participants or age and gait-speed matched controls.

Ankle stiffness and damping values were investigated for both the paretic and non-paretic limbs in the chronic stroke population as well as for healthy age-matched control subjects (Fig. 1). In subjects post-stroke, stiffness had a mean inter-subject variation of 1.5 ± 0.34 Nm/rad/kg for the paretic limb and 2.4 ± 0.23 Nm/rad/kg for the non-paretic limb. The repeated measures ANOVA comparing stiffness across timing point (30%, 50%, 70%, and 85% of stance phase) and limb (paretic, non-paretic) found stiffness varied significantly with respect to limb (*p* < 0.001, F_1,55_ = 29.6), but not timing point (*p* = 0.58, F_3,55_ = 0.67). The interaction between limb and timing point was not significance (*p* = 0.481, F_3,55_ = 2.39). For three older adults with no history of stroke, data were collected as age-range and gait speed matched reference for participants with chronic stroke, but were not included in statistical comparisons due low number of participants. Older adults walking at the same speed as chronic stroke participants exhibited a similar pattern of stiffness variation to young healthy adults^14,16^, but with a lower peak stiffness prior to heel off.

The estimated damping component of impedance had an inter-subject variability 0.008 ± 0.003 Nms/rad/kg for the paretic limb and 0.009 ± 0.005 Nms/rad/kg for the non-paretic limb. The repeated measures ANOVA for damping estimates found damping estimates did not vary significantly across timing point (*p* = 0.70, F_3,55_ = 0.48), or limb (*p* = 0.30, F_1,55_ = 1.08), but the interaction between these variables neared significance (*p* = 0.06, F_3,_ = 2.58). Post-hoc comparisons with Bonferroni corrections found damping varied significantly with limb only during the end of loading response/early mid-stance, 30% stance (*p* = 0.012, F_1,13_ = 12.49). Both the paretic and non-paretic limb lack the stereotypical increase in damping in preparation for toe off seen in the young, healthy adult. Ankle damping for older adults without stroke trended toward a larger magnitude when compared to the chronic stroke population across stance phase, which is notably increased compared to previous results for young adults walking at a faster pace^14,16^.

The average inertial component of impedance of the paretic and non-paretic limbs were 0.046 kg m^2^ and 0.0441 kg m^2^ respectively. Inertial results for each participant are summarized in Supplemental Information Table 2. The repeated measures ANOVA for inertia estimates showed no significant variation across timing point (*p* = 0.12, F_3,55_ = 2.07) or limb (*p* = 0.23, F_1,55_ = 1.47). There was no significant interaction between timing point and limb (*p* = 0.39, F_3,55_ = 1.02).

### Stiffness and damping relationship to EMG

**Figure 2:**
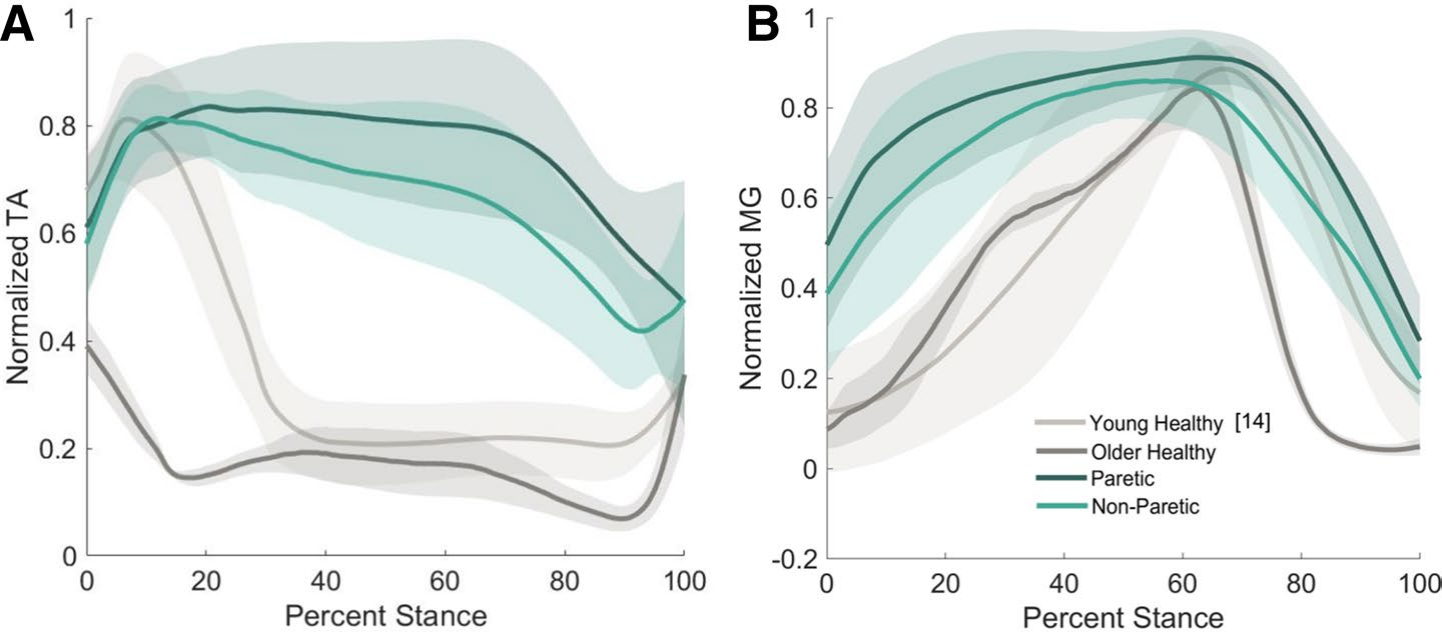
Average normalized EMG of the tibialus anterior (**A**) and medial gastrocnemius (**B**) across the stance phase of walking. Trials were normalized to the average peak EMG activity of a muscle throughout stance for each participant.

**Figure 3:**
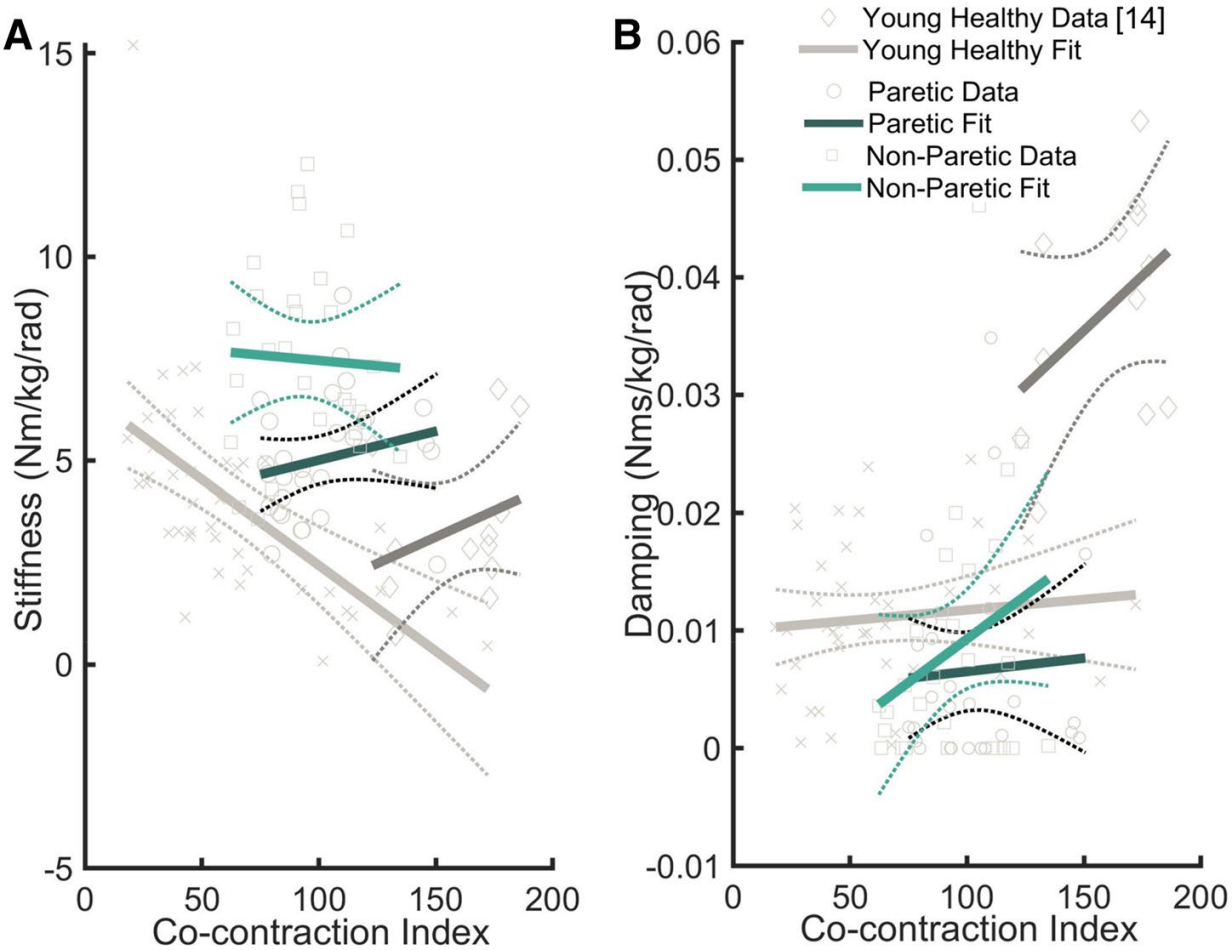
Stiffness (**A**) and damping (**B**) regressed across co-contraction index. Stiffness and CCI were significantly correlated for the paretic limb of individuals with chronic stroke and the young healthy adult; however the displayed opposite correlations. During walking, for the young healthy adult increased co-contraction was associated with lower stiffness, while for the paretic limb increased co-contraction was associated with higher stiffness. The non-paretic ankle stiffness of stroke participants and gait speed matched older adults did not correlate with co-contraction index. Ankle damping was not correlated with CCI.

Muscle electromyography (EMG) were recorded and investigated for trends (Fig. 2). To elucidate the relationship between the components of impedance and muscle co-contraction, stiffness and damping were each linearly regressed with Co-Contraction Index (CCI)^37^ (Fig. 3). The stiffness component of impedance demonstrated positive relationship with CCI for the paretic limb (slope = 0.014, *p* = 0.271, R^2^ = 0.0464) and a negative correlation with the non-paretic limb (slope = − 0.0053, *p* = 0.815, R^2^ = 0.0022), but neither significantly correlated. Ankle stiffness of gait-speed-matched older adults without a stroke also did not significantly correlate with CCI (slope = 0.02, *p* = 0.314, R^2^ = 0.1), while the young healthy limb demonstrated a significant negative correlation (slope = − 0.042, *p* < 0.001, R^2^ = 0.36). The damping component of impedance demonstrated positive relationships with CCI, but did not significantly correlate for any limb: paretic (slope = 2.3E−5, *p* = 0.755, R^2^ = 0.0038) non-paretic (slope = 1.5E−4, *p* = 0.145 R^2^ = 0.08), older adults (slope = 1.9E−4 *p* = 0.162, R^2^ = 0.19), or young adults (slope = 1.8E−6, *p* = 0.5144, R^2^ = 0.011). It is noted that although stiffness correlated with CCI of the young healthy adult, this model explains less than 36% of the variance.

### Stiffness and damping relationship to clinical measures

**Figure 4:**
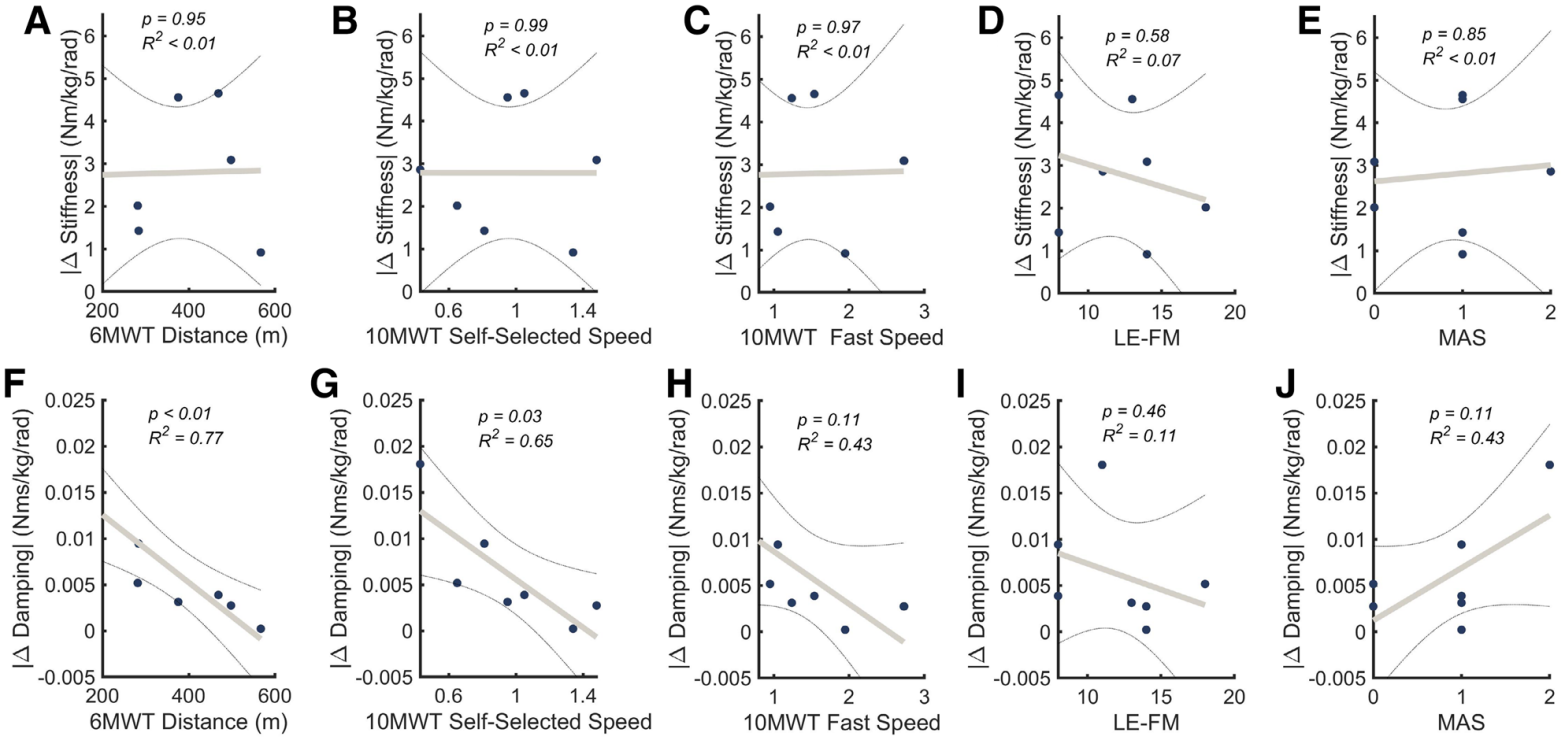
Stiffness (**A**–**E**) and damping (**F**–**J**) asymmetry linearly regressed across four clinical measures. Six Minute Walk Test distance was significantly correlated with the difference in damping between the paretic and non-paretic limbs (**F**), but did not relate to stiffness asymmetry (**A**). Ten Meter Walk Test speed was significantly correlated with damping asymmetry at the self-selected speed (**G**) but not the fast speed (**H**). Stiffness asymetry did not correlate with either 10MWT (**B**,**C**). Lower extremity Fugl–Meyer motor score did not significantly correlated with either ankle stiffness asymmetry (**D**) or damping asymmetry (**I**). Similarly, modified Ashworth score did not significantly correlate with either damping (**J**) or stiffness (**E**) asymmetry.

To investigate the relationship between changes in ankle impedance post-stroke and standard clinical measures used to characterize impairment, a series of linear regressions were performed (Fig. 4). Clinical measures for each chronic stroke participant are summarized in the Supplemental Information Table 1, with the metrics pertaining to the ankle used in analysis highlighted. The average absolute difference in damping across stance phase between the paretic and non-paretic limbs (damping asymmetry) and the 6MWT distance showed a significant negative correlation, and asymmetry predicted 77% of the variance in 6MWT distance (slope = − 3.66E−5, *p* = 0.0091, R^2^ = 0.774). Similarly, a significant correlation was found between the damping asymmetry and 10MWT speed for the self-selected speed (slope = − 0.013, *p* = 0.029, R^2^ = 0.649) but not for the fast speed (slope = − 0.0056, *p* = 0.111, R^2^ = 0.427). Damping asymmetry was not significantly correlated with the MAS score (slope = 0.0057, *p* = 0.0.11, R^2^ = 0.005), or the LE-FW motor score (slope = − 0.0006, *p* = 0.46, R^2^ = 0.113). The average absolute difference in stiffness between the paretic and non-paretic limbs did not significantly correlate with any clinical measure (*p* > 0.58).

A more detailed analysis of each timing point was performed for the aforementioned significant relationships. For each timing point, the signed difference in damping was regressed with the clinical measure of interest. This analysis showed damping asymmetry significantly correlated with 6MWT distance during powered push off at 70% stance (slope = 7.74.E−5, *p* = 0.0285, R^2^ = 0.65), and 85% stance (slope = 3.36.E−5, *p* = 0.0442, R^2^ = 0.59), but not during loading response and mid-stance (*p* > 0.156). Although the average absolute change in damping across stance correlated with the 10MWT self-selected speed, no individual timing point demonstrated a significant relationship. However, similar to the 6MWT, the relationship neared significance in late terminal stance (85% of stance: slope = 0.012, *p* = 0.0623, R^2^ = 0.53), but not at the other timing points (*p* > 0.08).

Finally, linear regressions were performed between clinical measures of mobility (6MWT and 10MWT) and clinical measures of impairment (MAS and LEFM) to assess how clinically defined impairment related to locomotor capacity. No significant correlation was found between 6MWT and LEFM (*p* = 0.534, R^2^ = 0.058), 6MWT and MAS (*p* = 0.5441, R^2^ = 0.055), 10MWT and LEFM (*p* = 0.5732*,* R^2^ = 0.0475), or 10MWT and MAS (*p* = 0.663, R^2^ = 0.0286).

### Removed data

Participants CVA02, CVA03, and CVA12 did not meet inclusion criteria and therefore did not complete the study. Participants CVA05 and CVA08 did not have enough trials with sufficient perturbation and model agreement necessary to reliably estimate impedance for any time point for either the paretic or non-paretic limb, so was not included in analysis. Removing trials due to insufficient perturbation is a common step of these methods^14,16,37^; in this study, 21% ± 8% of trials were removed across participants. Additionally, for individuals with chronic stroke, 53% ± 10% of remaining trials were removed due to poor model reliability (< 50% variance accounted for (VAF)). The distribution of stiffness estimates for included trials with good model fit (> 50% VAF) as well as for excluded trials with poor model fit (< 50% VAF) are included in Supplemental Information Fig. 1 for all participants who completed the experimental paradigm.

## Discussion

This purpose of this study was threefold: (1) to determine ankle mechanical impedance in individuals with chronic stroke during the stance phase of walking, (2) investigate how these properties relate to muscle activity, (3) quantify the relationship between mechanical impedance impairment, and clinical measures of mobility, spasticity, and sensorimotor function. We hypothesized that compared to the non-paretic and unimpaired joint, stiffness of the paretic ankle would be increased during loading response (early stance), where muscle activity is limited and passive stiffness dominates^33,34^, but decreased during mid-stance associated with reduced peak muscle activity^35^. As hypothesized, our results showed higher stiffness values at the beginning of stance phase for both the paretic and non-paretic limbs when compared to unimpaired participants. Additionally, paretic ankle stiffness was decreased in comparison to the non-paretic limb across stance phase, but this difference was not associated with differences in muscle activity. The damping component of impedance of the paretic and non-paretic limbs did not increase in preparation for toe-off, and did not significantly differ between limbs. Our secondary hypothesis was that clinical measures of mobility would correlate with impedance impairment during walking, but clinical measures obtained passively would not. The damping component of impedance correlated with mobility (6MWT and 10MWT), but the stiffness component of impedance did not. Furthermore, neither damping or stiffness impairment during walking correlated with clinical measures of spasticity obtained passively (MAS), or sensorimotor function (LE-FM).

### Impedance comparison between the paretic and non-paretic ankle

In early loading response and mid-stance the stiffness component of impedance of the paretic limb for participants with chronic stroke were similar in magnitude to results from passive impedance studies^20,22^. However, our results show an even greater stiffness increase of the non-paretic limb across stance phase, which was not found in impedance studies conducted in static postures. As hypothesized, paretic limb stiffness did not increase throughout mid stance and was significantly different from the non-paretic limb at the end of mid-stance and throughout terminal stance phase of walking (70% and 85% of stance). Reduced stiffness during mid-stance and early terminal stance phase has important implications for mobility of individuals with chronic stroke; during terminal stance phase, energy is added by the ankle joint. Increased overall stiffness of the non-paretic ankle during walking not present in passive conditions^20^ may reduce the energy able to be added by the ankle joint during walking.

The damping component of impedance did not vary across stance phase, and was not significantly different between paretic and non-paretic limbs. This aligns with impedance studies in static conditions that showed consistent damping across limbs following chronic stroke^20^.

Inertia estimates were consistent across stance phase and between limbs, but both the paretic and non-paretic ankle inertia estimates were increased relative to ankle–foot inertia reported in literature (0.015 kg m^2^^38^. Increased inertia estimates may indicate that the estimate represents the inertia of the foot and other coupled body segments or the inertia of the platform robot. Misalignment of the ankle and the robot’s centers of rotation could cause the perturbation to also effect displacements of local body segments and contribute to higher, more variable inertia estimates.

The contribution of each component of ankle mechanical impedance—stiffness, damping, and inertia—to model predicted torque response differed in chronic stroke participants from previous results in young healthy adults^14,16^. Torque contributions were variable across stroke participants, with the stiffness component of impedance contributing 30.3 ± 17.7% to ankle torque, compared to the 67% torque contribution seen in young healthy adults^14^. In participants with extremely low ankle stiffness, the contributions of damping and inertial components to torque were greater than the contributions of ankle stiffness.

### Impedance comparison between chronic stroke and unimpaired participants

Data were collected from three unimpaired older adults walking at similar speeds to elucidate how age may affect unimpaired ankle impedance values during gait. The stiffness component of impedance varied in a similar manner throughout stance phase to previous results in young healthy adult walking at a faster speed^14,16^. Stiffness increased to a peak around 70% of stance, then decreased during late terminal stance. Although, the peak ankle stiffness of the older adult was reduced when compared to the young healthy adult. Paretic ankle stiffness estimates of chronic stroke participants were increased during loading response and early mid-stance when compared to the gait-speed-matched older adults, however did not significantly vary across stance phase, and therefore did not display the stereotypical increase in stiffness during mid-stance phase. Non-paretic ankle stiffness estimates of chronic stroke participants were also increased when compared gait-speed-matched older adult, and reached a higher peak magnitude prior to powered push-off.

The damping component of impedance of the paretic and non-paretic ankles were similar in magnitude to those of the young unimpaired adult prior to heel off, but did not increase throughout late terminal stance in preparation for toe off. Notably, gait-speed matched older adults displayed a similar temporal variation to individual with chronic stroke, but a significantly higher magnitude than both the chronic stroke participants and young healthy adult. This may indicated that overall ankle damping increases with age, and reduced damping in both limbs of the chronic stroke population is an impairment. Alternatively, it is possible that higher damping estimate are a product of the difference in experimental methods. The ankle angle data of age-range-matched older adults was collected using motion capture, while participants with chronic stroke had their ankle data recorded using an electrogoniometer (consistent with previous works^14,16,39^). Future research should investigate how ankle damping is altered with in a larger cohort of older adults to formally isolate the effect of stroke on ankle damping.

### Impedance relationship to muscle activation

While, our hypothesis was supported that paretic ankle stiffness would decrease compared to the non-paretic ankle during mid stance and early terminal stance phase, the relationship to muscle activity is unclear. There was no difference in normalized muscle activity of the TA or MG muscles between the paretic and non-paretic limbs. However, the TA muscle activity did differ from that of gait-speed-matched older adult and the young healthy adult (Fig. 2). The CCI was regressed against ankle stiffness and damping to further investigate the relationship between muscle activity and impedance. These results align with studies investigating ankle stiffness when in static postures, which showed stiffness increases with activation and co-activation of the tibialis anterior and soleus muscles^25,29^. However, this directly contrasts with the relationships found in the young healthy adult during walking. Stiffness in this population was significantly negatively correlated with co-contraction index; meaning higher ankle stiffness was associated with lower levels of co-contraction. Furthermore, although the relationship was not significant, stiffness of the non-paretic ankle also displayed negative trend. A negative relationship in less intuitive, however, it does support the findings of Whitmore et al*.* which showed that changes in EMG tended to directly oppose the changes in stiffness when position and torque were continuously varying^32^. Specifically, during eccentric contraction, they found no appreciable change in plantarflexor EMG despite a large change in torque and stiffness^32^. Triceps surae muscles contract eccentrically when elastic energy is stored in preparation for push-off^40^, therefore a reduction in stiffness may not necessarily correspond with a reduction in EMG activity.

Without a significant reduction in EMG, one possible explanation for differences in limb stiffness relates to postural changes during walking exhibited by individuals post-stroke. During mid and terminal stance phase, individuals with chronic stroke often have reduced supination and increased pronation of the ankle–foot complex. Pronation of the foot is associated with eccentric contraction, reduced ankle stiffness and increased range of motion^41,42^. Increased pronation and eccentric contraction may, in part, explain the reduction in stiffness seen during mid stance. It is difficult to make conclusions regarding how stroke has altered the relationship between muscle activity, torque, position, and stiffness during walking since knowledge of these relationships is limited for the unimpaired population. However, our results underscore the importance of investigating walking directly, rather than extrapolating results obtained from postural tasks.

### Impedance relationship to clinical measures

#### Damping asymmetry correlates with measures of mobility

The difference in damping estimates between the paretic and non-paretic limbs significantly correlated with both measures of mobility (6MWT distance, and 10MWT self-selected speed), explaining 77% and 65% of the variance, respectively. However, damping asymmetry was not significantly correlated with 10MWT fast speed. The reduced correlation between ankle damping asymmetry and 10MWT fast speed when compared to the self-selected speed, might be explained by the redistribution of overall power contribution between joints at faster walking speeds. At slower walking speeds for a young unimpaired adult, the ankle contributes slightly more than the hip to positive power generation, while at faster walking speeds this relationship is switched^43^. Investigation of joint damping has been minimal, with most research focusing on joint stiffness. However, our results suggest that damping asymmetry may be a factor in reduced mobility of chronic stroke survivors. This result is contrary to previous work that found no significant correlation between passive ankle impedance and gait speed^19,44^, and highlights the importance of studying this property during dynamic tasks. Our results agree with Hsu et al. found that spatiotemporal gait asymmetry was primarily influenced by degree of spasticity^45^—a clinical description of altered joint impedance. Previous work also found gait asymmetries are closely related to dynamic balance in individuals with chronic stroke^46,47^*.*

#### Non-significant correlations

Modified Ashworth Scale (MAS) did not correlate with the difference in stiffness or damping between the paretic and non-paretic limb during walking. These results were expected since the MAS is evaluated while the subjects is relaxed and with an open kinematic chain, whereas impedance measurements occurred while muscles are actively contracting during walking, with a closed kinematic chain. Our results align with previous impedance studies in postural conditions, which also did not find a significant correlation between stiffness and MAS^19,48–50^ as well as a number of studies that have demonstrated that MAS do not relate to hyper reflexivity during walking in spastic gait^5,50,51^. This suggests that MAS may not be measuring impaired impedance of joints in a way that translates to descriptions of impairment during dynamic or functional tasks.

Asymmetry of either impedance parameter (i.e. stiffness and damping) also did not relate to the Lower Extremity Fugl Meyer (LE-FM) motor score. The motor score evaluates movement, coordination, and reflex action about the hip, knee, and ankle. These results suggest that, while useful in assessment of overall sensorimotor impairment, the LE-FM is not sufficient to characterize changes in mechanical impedance specifically. Therefore, it is unlikely that these changes have been addressed in clinical rehabilitation. However, it is important to note that the difference in stiffness also did not relate to clinical measures of mobility (6MWT, 10MWT), therefore focusing on correcting impaired ankle joint damping may be more beneficial for improving overall mobility for individuals with chronic stroke.

### Implications for patient care

The re-acquisition of the ability to walk community distances at reasonable speed post stroke is of critical importance for stroke survivors given the impact of walking on functional independence and cardiovascular health^4,52^. Currently, healthcare practitioners treating gait disorders in stroke survivors predominantly attend to the correction of sagittal plane swing phase errors with orthotic management or bracing. This emphasis is understandable given that excessive plantar flexion during mid to late swing phase is easily detected by patient and caregiver, and gives rise to variability in foot clearance which markedly increases risk of trips and falls^53,54^. However, the data we present suggest that stroke also changes the less easily detected mechanics of ankle-the foot complex during mid-stance and terminal stance phase of gait. These regions are especially important for energy storage and release^55^*,* and impairment to mechanical impedance properties could hinder forward propulsion, reducing gait speed and endurance. Our results show that following stroke, overall ankle stiffness is increased, but temporal variation is lost. The damping component of impedance also lacks the stereotypical increase during terminal stance. Furthermore, asymmetry in ankle joint damping between the paretic and non-paretic limb influence walking distance and speed to a greater extent than a validated composite measure of lower limb sensory and motor function. These results suggest that optimal rehabilitation of hemi-paretic stroke patients may require an expansion of clinical efforts to include the modification of stance phase changes in ankle–foot stiffness and damping.

Most pharmacological interventions used to treat swing phase kinematics also serve to diminish stance phase ankle–foot stiffness. Stiffness during mid-late stance is related to foot supination in mid-late stance, which is mediated primarily by the posterior tibialis muscle. However, during swing phase, posterior tibialis spasticity also leads to plantar flexion and inversion which increases trip risk. Consequently, this muscle is commonly injected with neurotoxin by clinicians, a decision commonly driven by observation of gait and/or clinical assessment with MAS. While the application of neurotoxin would be expected to similarly improve stiffness impairment during loading response (early stance) of walking, the data presented suggest that neurotoxin to the posterior tibialis muscle would be expected to further diminish ankle–foot stiffness in mid-late stance phase; thus improvements in walking speed and distance would be unlikely. These results agree with Lizma et al*.*, who found no improvements in gait speed or distance despite reported improvements in active ankle dorsiflexion, gait quality, and reduced spasticity as determined by MAS in their review of trials using neurotoxin to treat ankle plantar flexor and invertor muscles (always including the posterior tibilias muscle)^56^. Similarly, in their meta-analysis, Sun et al*.* reported neurotoxin-mediated improvements in LE-FM score but no improvement in gait speed^57^. Finally, it is unclear how pharmacological interventions affect ankle–foot damping, representing a major gap in knowledge given that our data suggests that damping impairment has significant impact on mobility.

Therefore, strategies to diminish swing phase plantar flexion and inversion post-stroke that do not reduce mid stance ankle–foot stiffness as well as strategies that address damping asymmetry are needed. Functional electrical stimulation is one such possibility. One of the few studies to show an increase in walking speed after neurotoxin therapy to the posterior tibilias muscle and plantar flexors combined neurotoxin with functional electrostimulation (FES) of the common peroneal nerve^58^. Further research is required to elucidate if FES can address ankle stiffness and damping impairments. In addition to studying the relationship between FES and joint impedance, future lines of research might also focus on a means to reduce hemi-paresis induced muscle spasticity without the contractile paralyzing effects of neurotoxins. Hyaluronidase therapy, which diminishes high impedance-inducing build-up of the compound hyaluronan in paretic muscles, is one such possibility^59^.

Finally, this research informs clinicians that ankle–foot stiffness and damping during stance phase of gait cannot be accurately gauged at the bedside using the Modified Ashworth Scale. Furthermore, the MAS did not appear to influence walking speed or distance in our participants. Therefore, although clinical strategies aimed at decreasing spasticity as determined by the MAS post stroke may serve to improve positioning or comfort, these strategies may not be expected to impact gait speed or distance. At this point there is no known bedside strategy for measuring or estimating ankle–foot stiffness and damping during the stance phase of gait in the setting of chronic stroke, which represents a further knowledge gap to be addressed in future research.

### Limitations

While this study provides first insight into ankle impedance in individuals with chronic stroke during walking, it assumes quasi–static second order dynamics during the analysis window surrounding each timing point (30%, 50%, 70%, 85% of stance phase). This is a simplification of actual ankle dynamics, which constantly vary throughout stance, however, this model was chosen for initial investigation of ankle impedance based on the previous success of this technique^14,16,60^. Additionally, impedance identification requires a perturbation to be applied to the joint, and therefore current technological limitations prevent the practical application of more sophisticated techniques during walking. While these assumptions proved successful in general for the population, a subset of trials in all individuals with chronic stroke did not reliably exhibit second order ankle dynamics. One possible explanation is that this methodology requires an isolated perturbation of the ankle joint during stance phase while the contralateral limb in in swing. Individuals with chronic stroke exhibit extremely heterogeneous gait patterns, sometimes exhibiting extended double support phase and excessive hip abduction, which would hinder joint isolation in some trials. It is also possible that the assumption of linear quasi-static behavior of the ankle is not maintained in the participants with more inconsistent gait mechanics. Future work should investigate the variability in joint dynamics of individuals post-stroke, and determine potential changes to the experimental methods that could account for this variability.

Despite efforts to control the ankle and platform robot’s center of rotation alignment, the location of heel contact on the platform varied across trials. Slight variations in each trial are expected using this methodology, especially when studying a population with gait inconsistencies. Previous studies quantified the sensitivity of stiffness estimates to misalignment, showing a 6% decrease in stiffness per cm misalignment^61^. The average intra-subject misalignment of rotation axes was 0.8 ± 4.9 cm for the paretic ankle, and 0.3 ± 3.0 cm for the non-paretic ankle. Therefore, this work predicts potential stiffness errors of 4.8 ± 29.4% and 1.8 ± 18% for the paretic and non-paretic ankles respectively.

For this initial investigation the chronic stroke population included was limited to nine individuals, all community ambulators, walking at a single gait speed. Even in this small subset of the population, impedance estimates were much more variable across participants than in unimpaired individuals. Therefore, extending the conclusions from this study on how impedance is altered following stroke to individuals with higher levels of impairment should be done with caution; altered ankle impedance post-stroke may differ with impairment level. It has also been shown that ankle impedance varies with gait speed in the young healthy adult population^39^, which is likely the case for the stroke population as well. Furthermore, statistical comparisons to gait-speed-matched older adults without history of stroke could not be made due to low number of older adult participants. Lower participant numbers stem from the long and arduous data collection process. Due to the lengthy protocol this study only characterized four timing points spanning the stance phase of walking. Future work should include data collected from additional participants, a broader range of post-stroke impairments, and other key gait events such as initial contact. Finally, while the methodology used in this study does accurately estimate overall ankle impedance during walking, it does not differentiate between intrinsic and reflex contributions to impedance. However, it is unlikely that our analysis characterizes ankle impedance values associated with reflexes (shown to be increased in some passive studies^20^) since sustained reflexes are minimized when using transient perturbations^60^ and the peak torque contribution from reflex activity occurs after a ~ 150 ms delay^62–64^, outside the 100 ms window of analysis used in this study.

## Materials and methods

### Participants

Twelve individuals with chronic hemiparetic stroke were enrolled in this study, nine of which completed the full protocol (5 male, 4 female, age 46 ± 9 years, weight 87 ± 15 kg, time since stroke 7.5 ± 2.5 years). All participants were required to have no history of major ankle injury or Botulinum Neurotoxin (BoNT) treatment for ankle spasticity, as well as being at least 2 years post-stroke. Individuals unable to complete the Six Minute Walk Test (6MWT) and those with a self-selected Ten Meter Walk Test (10MWT) speed less than 0.45 m/s were excluded. Three recruited participants were excluded: CVA02 was unable to complete the 6MWT without stopping, CVA03 had a self-selected walking speed as determined by the 10MWT below the required cut-off, and CVA12 opted not to complete the entire study. Additionally, three age-range matched adults were recruited (1 male, 2 female, age 57 ± 2 years, weight 62 ± 4.5 kg, termed “older adults” in this paper in order to distinguish from younger adults referenced from previous works. Approval for this study was granted by the Northwestern University Institutional Review Board and the University of Michigan Institutional Review Board, in accordance with all relevant guidelines. Prior to data collection, all participants provided written informed consent to take part in the study.

### Apparatus

A mechatronic platform, subsequently referred to as the Perturberator Robot, was used to apply perturbations to the ankle and record data. The Perturberator was recessed into a 5.25 m walkway and was capable of eliciting rotational perturbations in the sagittal plane. An AC gear motor (model: AKM42H-ANC2C-00, Kollmorgen, Radford, VA) controlled by a commercial servodrive (model: AKD-B00606, Kollmorgen, Radford, VA) was used to drive the Perturberator to the desired position during perturbations. Finally, a multi-axis force platform (model: 9260AA3, Kistler Inc, Amherst, NY) was rigidly attached to the Perturberator Robot to measure ground reaction forces (GRF). A schematic of the Perturberator Robot is included in Supplemental Information Fig. 2, and a more detailed description and validation of this device are provided in^61^.

### Experimental protocol

#### Clinical measures

A number of standard clinical measures were conducted to obtain a clinical metric of impairment for each chronic stroke participant, and to ensure that all participants had sufficient speed and endurance to participate. Two clinical measures of mobility were performed: the Six Minute Walk Test (6MWT), and the Ten Meter Walk Test (10MWT). The 6MWT assessed distance walked over six minutes as a submaximal measure of functional capacity in individuals post stroke. Walking speed and functional mobility over short distances was assessed using the 10MWT. Impairment level was assessed in participants meeting endurance and speed requirements using the Modified Ashworth Scale (MAS) and Lower Extremity Fugl Meyer (LE-FM). The MAS measured spasticity and each item was scored from 0 (no impairment) to 4 (severe impairment), while the LE-FM evaluated sensorimotor impairment, and with each item scored from 0 (severe impairment) to 2 (no impairment). Total MAS and LE-FM scores pertaining to the ankle joint were used to analyze the relationship to ankle impedance. The Spinal Cord Assessment Tool for Spastic Reflexes (SCATS) was collected as an additional measure of impairments for participants, but was not included in impedance analysis. All metrics were performed by the same licensed physical therapist.

#### Data collection

Data for participants with chronic stroke were collected at the Shirley Ryan AbilityLab, while age-range-matched older adults were collected at University of Michigan, therefore minor differences in protocol arise based on locational resources. All participants (9 chronic stroke, 3 older adults) walked across the walkway at a pace of 55–60 steps/min. To ensure step frequency consistency between trials, participants were asked to match the frequency of a metronome, and were given time to familiarize themselves with the task prior to data collection. Foot placement on the Perturberator Robot was monitored throughout the experiment, and the starting position of each participant was adjusted to ensure the ankle and Perturberator Robot’s centers of rotation aligned. Ankle angle was measured using electrogoniometers (ADInstruments, Inc. Sydney, AU) affixed to each ankle of chronic stroke participants, while ankle angle was measured using motion capture (model: Miqus M3, Qualisys AB, Gothenburg, Sweden) for older adult participants. GRFs during walking were measured using the force platform embedded in the Perturberator Robot. Electromyography (EMG) (Delsys, Natick, MA, USA) was collected from the tibialus anterior (TA), medial gastrocnemius (MG), semitendinosus (ST), and rectus femoris (RF) muscles of each leg under study (paretic and non-paretic limbs of chronic stroke participants). For older adults, EMG data were collected from the TA, MG, and Soleus muscles of the dominant limb. Each electrode site was cleaned with alcohol to facilitate electrode adherence and conduction of EMG signals. Electrodes were placed on the muscle belly parallel to the muscle fibers. All data were collected using 16-bit data acquisition systems (model: USB-6218/USB 2553, National Instruments, Austin, TX, USA) sampled at 1–2 kHz. As participants walked across the Perturberator Robot, a perturbation was randomly triggered with 50% probability. The Perturberator detected initial contact when the GRF passed the 50 N threshold. During trials that contained a perturbation, a delay timer was set based on walking speed for each time point to ensure the perturbation occurred at the desired location in stance phase. Specifically, ramp-and-hold perturbations occurred at approximately 30%, 50%, 70% or 85% of stance phase for chronic stroke participants, and at 30%, 45%, 65%, 85% of stance for older adults. For chronic stroke participants, 100 perturbation trials were collected for each timing point, and 30 perturbation trials were collected for older adults at each timing point. Perturbations were 2° (0.035 rad) in magnitude; however, it is noted that due to increased ankle stiffness and/or motion of the mid-foot, a 2° perturbation of the robot may not translate to a full 2° perturbation of the ankle joint. Finally, to mitigate slippage and prevent falls throughout the experiment, individuals post-stroke wore a safety harness secured to an overhead gantry system and treaded hospital socks (Medichoice, Mechanicsville, VA, USA).

### Analytical protocol

#### EMG analysis

For each participant, EMG data for all trials were processed prior to dividing data into steps. EMG data were bandpass filtered (50–200 Hz) and full wave rectified. Then a 200 ms moving average filter was applied to the rectified EMG. EMG data for each muscle were divided into steps and normalized to the average peak EMG across non-perturbed steps. Normalized EMG data were used to compare EMG activity between the paretic and non-paretic limbs across participants.

To investigate the relationship between the components of impedance and muscle activity, binned EMG at each timing point (30%, 50%, 70%, 85% of stance phase) were determined by averaging non-normalized EMG for each participant in a 100 ms window beginning with the onset of perturbation (the same analysis window used to determine ankle impedance). A co-contraction index (CCI) was then determined for muscles spanning the ankle each participant at each timing point (30%, 50%, 70%, 85% of stance) using this averaged EMG data.

1 $$CCI=2\times \left(\frac{antagonist}{antagonist+agonist}\right)\times 100$$

During stance phase, the TA is the antagonist muscle, and the MG is the agonist muscle.

#### Impedance analysis

Trials in which the ankle displacement perturbation was less than 0.75° or were not included in analysis to ensure sufficient perturbation in the presence of data variability. Trials were also excluded if second order model explained less than 50% of variance. Data for these trials are included in Supplemental Information Fig. 1, but were excluded from the main results due to poor signal to noise ratio or uncertainty of impedance estimates.

Force plate data, motor encoder data, and ankle goniometer data were low-pass filtered using a bidirectional third-order Butterworth filter with a 20 Hz cutoff frequency. Forces arising from the Perturberator Robot’s intrinsic inertia were removed using previously estimated linear filters^61^. Data were then divided into steps and separated into paretic limb and non-paretic limb. Sagittal plane ankle torque was determined by resolving GRFs to the equivalent force-torque couple at the ankle’s center of rotation:

2 $$T= {F}_{z}{d}_{x}+{F}_{x}{d}_{z}$$

where *F*_*x*_ and *F*_*z*_ are the anterior–posterior and vertical GRF respectively, and *d*_*x*_ and *d*_*z*_ are the corresponding moment arms. Moment arms were determined by transforming center of pressure data to the ankle frame of reference. When the foot is flat on the ground during at the end of loading response (early stance), during mid-stance phase, and during terminal stance prior to heel off, the anterior–posterior distance from the COP at heel contact to the ankle frame was subtracted from COP data. During late terminal stance, as the heel rises, the mid-foot deforms and a more complex transformation is required. A biomechanical model of the foot, was used to transform COP data to the ankle frame while accounting for movement of mid foot segments^16^.

Due to the heterogeneous nature of steps in the chronic stroke population, data were converted to a phase based representation of stance, such that key features of stance phase align across trials. For each limb (paretic, non-paretic and older adult), perturbation trials were separated from non-perturbation trials, then further segmented into the four perturbation timing points of interest (30%, 50%, 70% and 85% stance phase or 30%, 45%, 65% and 85%, respectively). For older adults, segmented data were bootstrapped in accordance with previously published methods for estimating impedance^14,16^ to provide an estimate of variability. The initial trial in each bootstrap was selected at random, and a probability algorithm selected additional trials such that temporally-similar trials were selected with higher probability until 60% of trials for a specific time point. This method was used to account for slight differences in timing of perturbations at each time point. Offset was removed such that both torque and angle begin with zero. Bootstrapped ankle angle and torque arising naturally during walking (non-perturbed trials) were subtracted from perturbation trials in order to isolate the perturbation response, then trimmed to 100 ms windows beginning at the onset of perturbation.

Individuals with chronic stroke exhibited highly variable steps such that consistent trends in the non-perturbed data could not be established. Thus, previous methods were modified to address the heterogeneous nature of post-stroke kinematic and kinetic data. Rather than the bootstrapping procedure used in previous studies, we estimated impedance properties for each perturbed trial. For each perturbation trial, the perturbation was isolated by subtracting the average ankle angle and torque arising naturally during walking from the fifty non-perturbed trials that most resembled that perturbation trial. The 50 non-perturbed trials that maximize kinetic and kinematic agreement (“Fit” as defined by (3)) were selected as non-perturbed trials for a specific perturbation trial.

3 $$Fit=\frac{{VAF}_{angle}+{VAF}_{torque}}{2}$$

where VAF_angle_ is the variance accounted for between the perturbed trial’s ankle angle and a non-perturbed trial’s ankle angle excluding the perturbation window, and VAF_torque_. is the variance accounted for between the perturbed trial’s ankle torque and a non-perturbed trial’s ankle torque excluding the perturbation window. Data were then trimmed to 100 ms windows beginning at the onset of perturbation Ankle impedance was estimated over the 100 ms window of each trial using least-squares system identification. A second order parametric model mapped perturbation induced displacement to the resultant torque response at the ankle.

4 $$T=I\ddot{\theta }+b\dot{\theta }+k\theta$$

where *T* and *θ* are the torque and angle response arising from the perturbation respectively, *I* is the total inertia of the foot–ankle complex, *b* is the damping component of ankle impedance, and *k* is the stiffness component of ankle impedance. Angular velocity and acceleration were determined numerically by differentiating the ankle angle data^65^.

### Statistics and comparisons

#### Impedance estimates

The primary aim of this study was to characterize the effect of chronic stroke on impedance during the stance phase of walking. Three repeated measures analysis of variances (ANOVA) were performed in which stiffness, damping and inertia were dependent variables. Timing point (30%, 50%, 70% and 85% of stance phase) and limb (paretic and non-paretic) were treated as fixed factors, and subject was treated as a random factor. The interaction between timing point and limb was examined. A significance level of α = 0.05 was set a priori; Bonferroni corrections were applied for multiple comparisons. Data from gait-speed matched older adults and young adults walking at a faster speed^14,16^ were included for reference, but statistical comparisons with the stroke population were not conducted.

#### EMG analysis

To investigate the source of changes in impedance post-stroke, the relationship between muscle activity and stiffness was examined for chronic stroke participants (paretic and non-paretic limb), gait-speed matched older adults, and young adults by fitting a linear regression between stiffness (measured at each time point) and co-contraction index (CCI). This analysis was repeated for the damping component of impedance.

#### Clinical measures

The secondary aim of this study was to evaluate the relationship between changes in impedance and standard outcome measures used to characterize impairment in the clinic. ΔStiffness scores were calculated for each participant and linearly regressed on each clinical measure (6MWT, 10MWT, LEFM, MAS). For each limb, stiffness as a function of percent stance phase was determined using an interpolation. A ΔStiffness score was then defined as the average absolute difference in stiffness between the paretic and non-paretic limbs across the stance phase. The same protocol was used to define ΔDamping scores.

5 $$\Delta Impedance=mean\left(\left|{f}_{p}-{f}_{np}\right|\right)$$

Impedance impairment post-stroke varied throughout stance phase such that in some portions of stance the paretic limb exhibited increased impedance, while in others stiffness and damping were reduced. The absolute change was selected to capture overall difference from the non-paretic limb. For comparisons that yielded a significant relationship between overall difference in an impedance parameter and a clinical metric, a more detailed analysis was conducted at each timing point separately. In these cases, the signed difference in stiffness between paretic and non-paretic limbs was linearly regressed on clinical measures.

## Supplementary Information

Supplementary Information.
