# Supplementary material for: Characterization and clinical implications of ankle impedance during walking in chronic stroke
Source: Sci Rep. 2021 Aug 18;11:16726. doi: 10.1038/s41598-021-95737-6 (PMC8373915; doi:10.1038/s41598-021-95737-6)
Supplement: Supplementary file 1 — Supplementary Information. [file 41598_2021_95737_MOESM1_ESM.docx]

# Supplementary Materials

*Table 1: Clinical Measures*

| **Clinical Measure** | **Subject** | | | | | | | | |
| --- | --- | --- | --- | --- | --- | --- | --- | --- | --- |
|  | ***CVA01*** | ***CVA04*** | ***CVA05*** | ***CVA06*** | ***CVA07*** | ***CVA08*** | ***CVA09*** | ***CVA10*** | ***CVA11*** |
| 6 Minute Walk Test |  |  |  |  |  |  |  |  |  |
| *Distance (m)* | 497.8 | 566.9 | 199 | 281.6 | 375.5 | 395.6 | 160.9 | 283.8 | 468.6 |
| *Exertion* | 6 | 13 | 7 | 11 | 13 | 13 | 13 | 7 | 14 |
| 10 Meter Walk Test |  |  |  |  |  |  |  |  |  |
| *Self-selected velocity (m/s)* | 1.48 | 1.34 | 0.53 | 0.65 | 0.95 | 1.14 | 0.43 | 0.812 | 1.05 |
| *Fast velocity (m/s)* | 2.73 | 1.95 | 0.95 | 0.95 | 1.24 | 1.7 | 0.74 | 1.054 | 1.54 |
| SCATS^b^ |  |  |  |  |  |  |  |  |  |
| *Plantar flexor clonus* | 0 | 0 | 0 | 0 | 0 | 2 | 3 | 0 | 1 |
| *Knee/Hip flexor spasms* | 1 | 0 | 0 | 0 | 1 | 1 | 0 | 0 | 0 |
| *Knee/Hip extensor spasms* | 0 | 0 | 0 | 0 | 0 | 0 | 0 | 0 | 1 |
| Modified Ashworth Scale ^c^ |  |  |  |  |  |  |  |  |  |
| *Hip flexors* | 1 | 0 | 1 | 0 | 0 | 0 | 0 | 0 | 0 |
| *Hip extensors* | 0 | 0 | 0 | 0 | 0 | 0 | 0 | 0 | 1 |
| *Hip Adductors* | 1 | 0 | 0 | 0 | 0 | 1 | 1 | 0 | 1 |
| *Knee flexors* | 1 | 1 | 2 | 0 | 1 | 1 | 1 | 1 | 1 |
| *Knee extensors* | 0 | 0 | 0 | 0 | 0 | 0 | 0 | 0 | 0 |
| *Dorsiflexors* | 0 | 0 | 0 | 0 | 0 | 0 | 0 | 0 | 0 |
| *Plantarflexors* | 0 | 1 | 3 | 0 | 1 | 2 | 2 | 1 | 1 |
| LE Fugl-Meyer ^d^ |  |  |  |  |  |  |  |  |  |
| *Motor function (28)* |  |  |  |  |  |  |  |  |  |
| Seated knee flexion | 2 | 2 | 2 | 2 | 2 | 2 | 1 | 1 | 2 |
| Seated dorsiflexion | 1 | 2 | 0 | 2 | 2 | 1 | 1 | 1 | 0 |
| Tremor | 2 | 1 | 2 | 2 | 2 | 1 | 2 | 2 | 2 |
| Dysmetria | 2 | 1 | 0 | 2 | 1 | 1 | 0 | 0 | 0 |
| Speed | 1 | 0 | 1 | 2 | 1 | 2 | 0 | 0 | 0 |
| Hip flexor synergy | 2 | 2 | 2 | 2 | 2 | 2 | 2 | 2 | 2 |
| Knee flexor synergy | 2 | 2 | 2 | 2 | 2 | 2 | 2 | 2 | 2 |
| Dorsiflexion synergy | 2 | 2 | 1 | 2 | 2 | 1 | 2 | 1 | 1 |
| Hip extensor synergy | 2 | 2 | 2 | 2 | 2 | 2 | 1 | 2 | 1 |
| Adductor synergy | 2 | 2 | 2 | 2 | 2 | 2 | 2 | 2 | 1 |
| Knee extensor synergy | 2 | 2 | 2 | 2 | 2 | 2 | 2 | 2 | 2 |
| Plantar flexion synergy | 1 | 2 | 1 | 2 | 2 | 2 | 1 | 1 | 1 |
| Knee flexion out of synergy | 1 | 2 | 0 | 2 | 1 | 1 | 1 | 1 | 0 |
| Dorsiflexion out of synergy | 0 | 1 | 0 | 2 | 0 | 0 | 1 | 0 | 0 |
| *Reflex Activity (4)* |  |  |  |  |  |  |  |  |  |
| Achilles | 2 | 2 | 2 | 0 | 0 | 0 | 2 | 2 | 2 |
| Patellar | 2 | 2 | 2 | 2 | 2 | 0 | 2 | 2 | 2 |
| *S*ensory Function (12) |  |  |  |  |  |  |  |  |  |
| Thigh light touch | 2 | 1 | 1 | 2 | 2 | 1 | 0 | 0 | 1 |
| Sole of foot light touch | 1 | 1 | 1 | 2 | 1 | 1 | 1 | 0 | 1 |
| Hip proprioception | 2 | 2 | 2 | 2 | 2 | 2 | 2 | 2 | 2 |
| Knee proprioception | 2 | 2 | 2 | 2 | 2 | 2 | 2 | 2 | 2 |
| Ankle proprioception | 2 | 2 | 2 | 2 | 2 | 2 | 1 | 2 | 2 |
| Toe proprioception | 2 | 2 | 0 | 2 | 2 | 2 | 1 | 1 | 1 |
| 1. Clinical measures pertaining to the ankle are highlighted in green. 2. Scored from 0 to 2 where 0 indicates no impairment and 2 indicates severe impairment 3. Scored from 0 to 4 where 0 indicates no impairment and 4 indicates severe impairment 4. Scored from 0 to 2 where 2 indicates no impairment and 0 indicates severe impairment | | | | | | | | | |

*Table 2: Mean and standard deviation of inertia estimates for each participant at each stance time*

|  | | **Inertia (kgm^2^)** | | | |
| --- | --- | --- | --- | --- | --- |
| **Subject** | **Limb** | *30%* | *50%* | *70%* | *85%* |
| CVA01 | Paretic  Non-Paretic | 0.045±0.03  0.069±0.06 | 0.055±0.03  0.050±0.02 | 0.043±0.02  0.053±0.03 | 0.048±0.03  0.080±0.04 |
| CVA04 | Paretic  Non-Paretic | 0.011±0.03  0.010±0.03 | 0.068±0.08  0.024±0.04 | 0.069±0.06  0.078±0.07 | 0.099±0.06  0.075±0.06 |
| CVA06 | Paretic  Non-Paretic | 0.063±0.04  0.030±0.03 | 0.052±0.04  0.065±0.04 | 0.046±0.04  0.088±0.06 | 0.047±0.02  0.057±0.05 |
| CVA07 | Paretic  Non-Paretic | 0.093±0.04  0.008±0.01 | 0.089±0.05  0.021±0.04 | 0.091±0.001  0.057±0.05 | 0.056±0.04  0.001±0.0001 |
| CVA09 | Paretic  Non-Paretic | 0.068±0.06  0.061±0.09 | 0.11±0.09  0.12±0.09 | 0.095±0.1  0.10±0.08 | 0.062±0.05  0.11±0.05 |
| CVA10 | Paretic  Non-Paretic | 0.056±0.05  0.041±0.03 | 0.033±0.03  0.03±0.02 | 0.056±0.04  0.030±0.03 | 0.021±0.02  0.074±0.04 |
| CVA11 | Paretic  Non-Paretic | 0.046±0.05  0.012±0.02 | 0.040±0.03  0.024±0.03 | 0.061±0.06  0.041±0.05 | 0.070±0.07  0.062±0.03 |

*Figure 1: Distribution of stiffness estimates for each stance time (30%, 50%, 70%, and 85% stance) of included trials for which the second order model performed well (>50% VAF) are shown in blue. Distribution of excluded trials with poor model fit are shown in grey. Stiffness estimates are included for both the paretic (left) and non-paretic (right) limb of each participant that completed the entire experimental paradigm. Participants CVA05 and CVA08 were not included in the main analysis dud to an insufficient number of acceptable trials (>50% VAF)*


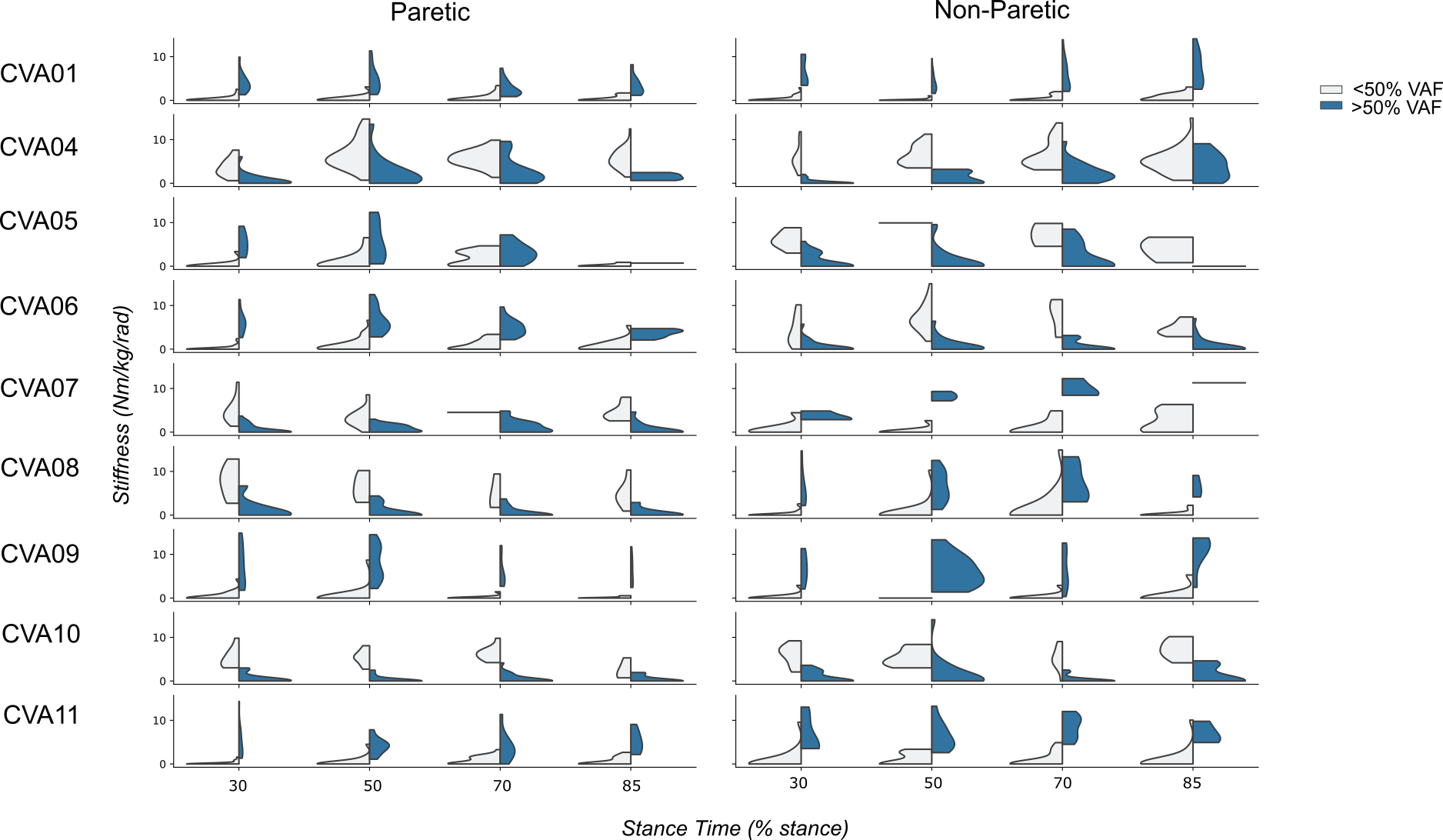


*Figure 2: Schematic of Perturberator Robot and experimental setup modified from [13].*


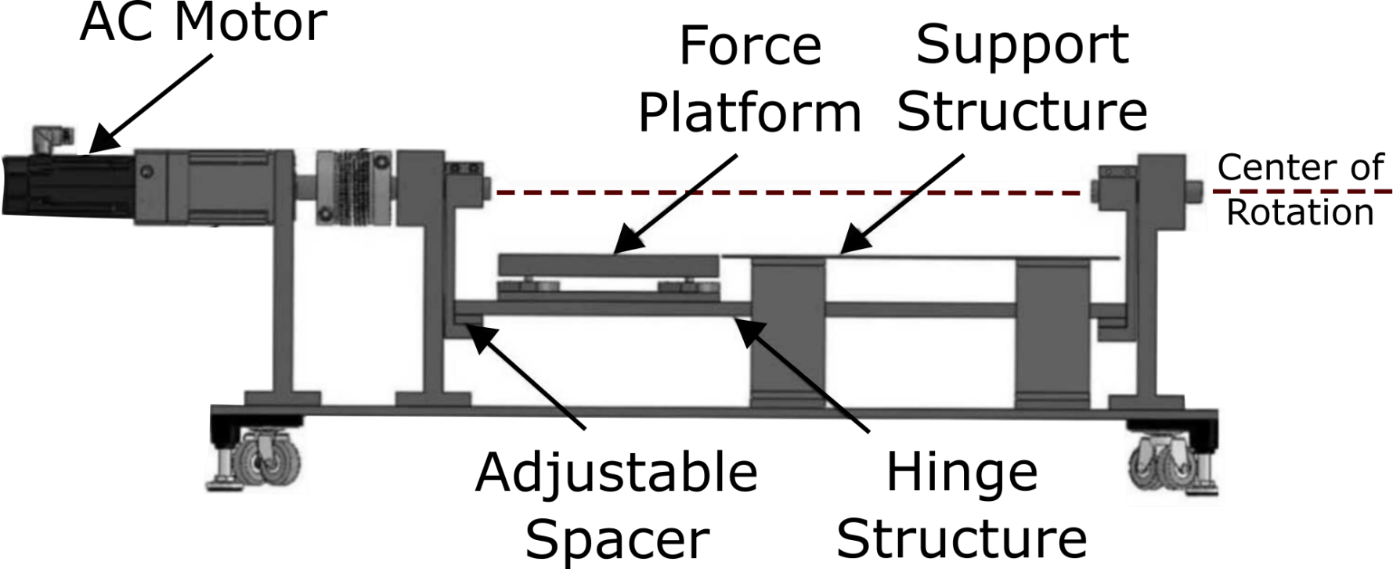


*Frontal Plane View*

*Sagittal Plane View*


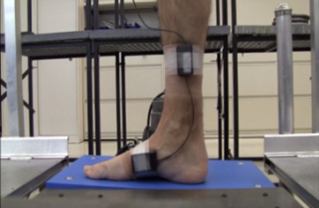

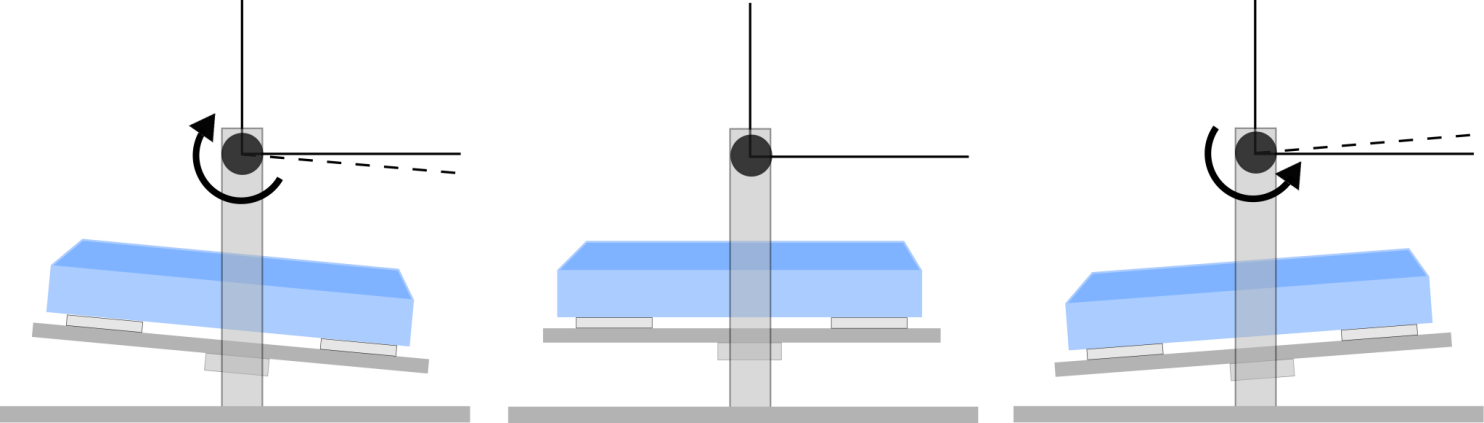


2◦ Dorsiflexion
